# Acute Pain Services and pain-related patient-reported outcomes in Hungarian hospitals

**DOI:** 10.1186/s13741-024-00373-z

**Published:** 2024-03-12

**Authors:** Orsolya Lovasi, Péter Gaál, Krisztián Frank, Judit Lám

**Affiliations:** 1 School of PhD Studies, Semmelweis University, Üllői Út 26, 1085 Budapest, Hungary; 2 Health Services Management Training Center, Semmelweis University, Budapest, Hungary; 3 Department of Applied Social Sciences, Sapientia Hungarian University of Transylvania, Targu Mures, Romania; 4 Szekszárd District Office of the Government Office of Tolna County, Szekszárd, Hungary; 5 NEVES Society for Patient Safety, Budapest, Hungary

**Keywords:** Acute pain service, Patient satisfaction, Pain assessment, Patient outcomes

## Abstract

**Background:**

Postoperative pain management is an important part of surgical care, where Acute Pain Service offers added value in terms of patient outcomes and costs. The technology, however, has hardly been adopted in Hungary, with only two hospitals operating Acute Pain Service and whose performance has not been evaluated yet. This research compared pain management outcomes of surgical, orthopedic, and traumatology patients in Hungarian hospitals with and without Acute Pain Service.

**Methods:**

We recruited 348 patients, 120 in the APS group and 228 in the control group, whose experience was surveyed with an adapted version of the American Pain Society Patient Outcome Questionnaire. The questionnaire covered pain intensity, pain interference with physical and emotional functions, side effects, patient satisfaction, information received, and participation in treatment decisions. The differences were analyzed by Fisher’s exact test and Mann–Whitney *U* test.

**Results:**

The APS group showed better results with lower pain intensity scores regarding worst postoperative pain (*χ*^2^ = 18.919, *p* = 0.0043). They reported less pain interference with activities in bed (*χ*^2^ = 21.978, *p* = 0.0006) and out of bed (*χ*^2^ = 14.341, *p* = 0.0129). Furthermore, patients in the APS group experienced fewer pain-management-related side effects, like nausea (*χ*^2^ = 15.240, *p* = 0.0101), drowsiness (*χ*^2^ = 26.965, *p* = 0.0001), and dizziness (*χ*^2^ = 13.980, *p* = 0.0124). However, patient information (*χ*^2^ = 3.480, *p* = 0.0945) and patient satisfaction (*χ*^2^ = 5.781, *p* = 0.2127) did not differ significantly between the two groups.

**Conclusions:**

Our findings confirm earlier international evidence on the benefits of Acute Pain Service in postoperative pain management and support the wider adoption of the technology in Hungarian hospitals. Nevertheless, close attention should be paid to patient information and involvement as better outcomes alone do not necessarily increase patient satisfaction.

## Background

Although significant progress has been made, achieving optimal postoperative pain management has been challenging for healthcare providers for decades (Sinatra 2010, Ahmed & Yasir 2015, van Boekel et al. 2015, Erlenwein et al. 2016, Zaccagnino et al. 2017). Inadequate pain relief is documented to increase morbidity, mortality (Meissner et al. 2008; Torabi Khah et al. 2020), drug consumption, medical treatment costs (Hayes & Gordon 2015, Petti et al. 2018; Pozek et al. 2018), the number of hospital readmissions, and the associated costs of reduced capacity to work (Pozek et al. 2018; Torabi Khah et al. 2020), while it is also detrimental to patient safety and decreases patient satisfaction (Torabi Khah et al. 2020).

The quality of postoperative pain treatment may improve in those hospitals, where, along with the deployment of up-to-date anesthesiology techniques and drugs, pain management is organized and operated by a multidisciplinary team of health professionals, the so-called Acute Pain Service (APS) (Stamer et al. 2002; van Boekel et al. 2015, Webb & Kim 2018). Better patient outcomes (Kersting et al. 2020), including reduced pain intensity (Lee et al. 2010; Fang et al. 2021), decreased anxiety about pain (Jepegnanam et al. 2021), and declining opioid consumption (Edwards et al. 2020; Stamer et al. 2020), have been reported. The uptake of the technology seems to be associated with the more frequent use of up-to-date pain relief procedures, such as patient-controlled analgesia (PCA) (Tawfic et al. 2021).

The technology has been pioneered by Ready et al. (Ready et al. 1988), and many hospitals followed suit worldwide (Stamer et al. 2020). Although the per capita costs of pain relief were found to be higher, APS could work more efficiently (Lee et al. 2010) as a result of early mobilization, early discharge, and better patient outcomes (Deni et al. 2019). The actual costs of care, however, depend on the organizational culture, the type of surgical patients attended, and the payment system in place (Sun et al. 2010).

Pain teams could be operated by an anesthesiologist (Miaskowski et al. 1999; Said et al. 2018), but there are also nurse-based and anesthesiologist-supervised teams (Goldberg et al. 2017; Edwards et al. 2020). Anesthesiologist residents (Borracci et al. 2016), and certified registered nurse anesthetists could also be involved (Edwards et al. 2020; Fang et al. 2021). As the technology evolved, the involvement of other health professionals, such as pharmacists, surgeons, physiotherapists, psychiatrists, and psychologists, has also been recommended by various guidelines (Sinatra 2010; Upp et al. 2013; Rockett et al. 2017). Nevertheless, the structure and composition of APS can vary depending on the health system context (Sinatra 2010; Stamer et al. 2020; Tawfic et al. 2021). An APS should provide a 24/7 service, develop pain management protocols, train the health care staff and patients, and regularly assess and document pain as well as organize clinical audits on a regular basis (Stamer et al. 2002, 2020; Upp et al. 2013).

As far as Hungary is concerned, the topic has remained mostly unexplored until recently (Lovasi et al. 2020). Our previous research has shown that the technology has hardly gained ground in the Hungarian health system with only two hospitals having reported an established APS, so far (Lovasi et al. 2021). This study takes one step further by evaluating the performance of these hospitals in terms of the postoperative pain relief of surgical patients compared to a control group, whose members were attended to in the traditional setting. The main objective of the research is to establish whether there is any difference between patient outcomes in hospitals with and without APS. It is important to note in advance that our results should be interpreted in the current context and challenges of the Hungarian health system (Gaál 2005; Szigeti et al. 2019; Szócska et al. 2021).

## Methods

### Study design

We conducted a comparative study among hospitals with and without APS to detect any differences in patient outcomes of surgical cases from the general surgical, orthopedic, and traumatology departments. The head nurses of each department were verbally informed about the study and were asked to hand over questionnaires to 40 selected patients per department, 24 h after surgery. The nurses distributing and collecting the questionnaires were informed both verbally and in writing about the aims of the survey and the inclusion and exclusion criteria of patients to be surveyed. Patients meeting the inclusion criteria were enrolled in the study consecutively until reaching the predetermined numbers. All the recruited patients received written information about the aims and process of the survey with phone numbers and e-mail addresses of the investigators. Questionnaires were only included in the study if the patient met the inclusion criteria and had given voluntary informed consent. Ethical approval was obtained from the Scientific and Research Committee of the Medical Research Council (approval number: IV/7038–2/2020/EKU).

Native Hungarian speaking patients aged 18 years or over, with a scheduled operation of general abdominal surgery, thoracic surgery, breast surgery, and orthopedic or trauma surgery at one of the aforementioned departments of the included hospitals, who were conscious and responsive during the involvement process and had no communication obstacles were eligible to participate in the study. Patients with cognitive impairment or dementia, patients needing intensive care or being sedated after surgery, and patients requiring emergency surgery were excluded.

### The questionnaire

Unidimensional scales measure only the intensity of pain, therefore, whenever the circumstances allow, multidimensional scales should be chosen (Radnovich et al. 2014; Petti et al. 2018, Lovasi et al. 2022), such as the American Pain Society Patient Outcome Questionnaire (APS-POQ), which has already been applied in the case of general surgery, urology, orthopedics, obstetrics, and traumatology patients (Dihle et al. 2008, Brown et al. 2013, Zoëga et al. 2014, Botti et al. 2015, O’Donnell 2015, Subramanian et al. 2016, Erden et al. 2018, Eshete et al. 2019) in the USA (Gordon et al. 2010), Denmark and Australia (Botti et al. 2015), China (Wang et al. 2017), Turkey (Erden et al. 2018), Norway (Dihle et al. 2008), and Malaysia (Subramanian et al. 2016), albeit a Hungarian version has not been validated yet. In most cases, the questionnaire can accurately measure postoperative pain; however, its use is not recommended for sedated, demented, non-communicative adults and children (Gordon et al. 2010, Lovasi et al. 2022).

The survey was conducted using an adapted version of the APS-POQ. Its translation, language validation, and adaptation were carried out by a team of anesthesiologists, surgeons, psychologists, and registered nurses. The questionnaire was piloted among 20 patients, who did not indicate any problems with interpretation.

The questionnaire covered the following areas: (1) pain intensity and relief, (2) the interference of pain with physical activity and sleeping, (3) the interference of pain with the emotional state of the patients, (4) pain-management related side effects, (5) participation in treatment decisions, and (6) non-pharmacological treatment strategies. Questions were rated on a scale from 0 to 10 or from 0 to 100%.

### Data collection

All the data were collected between 1 September 2020 and 15 March 2021. The ongoing COVID-19 pandemic complicated the data collection process, as the capacity of hospitals for elective surgical procedures had been limited to free up beds for COVID-19 patients. Due to a lack of dedicated research personnel, the head nurses handed out paper-based questionnaires after informing the patients about the study and obtaining their informed consent to participate. Patients filled out the questionnaires without any help from the personnel. Participation was voluntary and anonymous, without any financial compensation. We recorded the respondents’ age, sex, and socioeconomic status, the department where the operation was performed, the number of previous surgeries, and the APS-POQ scores, as described before. The collected data were recorded in an electronic database for further processing and analysis.

### Data analysis

During the data analysis, we compared the responses of the patients receiving care under APS (APS group) with the responses of patients, who were attended in the traditional setting (control group). The obtained scores on the point and % scales were converted further into five categories: none (0 or 0%), mild (1–3 or 10–30%), moderate (4–6 or 40–60%), severe (7–9 or 70–90%), and extreme (10 or 100%).

Qualitative data were described in terms of frequencies (number of cases), relative frequencies (percentages), and score distributions. Quantitative data were expressed as mean with standard deviation (± SD). The APS and control groups were compared in terms of frequencies and scores using the Fisher’s exact test and the Mann–Whitney *U* test, respectively. All statistical calculations were done with the help of SPSS 25 (IBM, Armonk, NY, USA). Significance values were corrected using the method of Benjamini and Hochberg (Benjamini & Hochberg 1995), and corrected “*p*” values lower than 0.05 were considered statistically significant.

## Results

A total of 348 completed questionnaires were included in the study. The APS group had 120 patients from hospitals operating an APS, while the control group had 228 patients with no APS. The two groups were comparable in terms of socioeconomic characteristics. Most patients were female, married or living with a partner, had completed secondary school education, and had at least one previous surgery in both groups (Table 1). The only statistically significant difference between the two groups was the level of education: the proportion of those with the lowest level of education was significantly higher in the APS group (*χ*^2^ = 10.527, *p* = 0.0146).

**Table 1:** Socioeconomic characteristics of recruited patients

Variable		APS group (*n* = 120)	Control group (*n* = 228)	*p*
Age		60.4 ± 16.6	58.3 ± 14.1	*U* = 11,524 *p* = 0.0992
Gender	Male	44.2% (53)	41.3% (94)	*χ*^2^ = 0.773 *p* = 0.6796
	Female	55.8% (67)	58.3% (133)	
	Unknown	-	0.4% (1)	
Education	Primary	31.7% (38)	17.5% (40)	*χ*^2^ = 10.527 *p* = 0.0146
	Secondary without graduation	27.5% (33)	30.7% (70)	
	Secondary with graduation	27.5% (33)	39.5% (90)	
	College/university	13.3% (16)	12.3% (28)	
Marital status	Single	14.2% (17)	11.0% (25)	*χ*^2^ = 3.009 *p* = 0.5564
	Married or living with partner	55.0% (66)	63.2% (144)	
	Divorced	10.0% (12)	7.4% (17)	
	Widowed	20.8% (25)	18.0% (41)	
	Unknown	-	0.4% (1)	
Previous surgeries		2.8 ± 2.4	2.4 ± 2.3	*U* = 12,255 *p* = 0.1339

Pain intensity was significantly higher in the control group, with 92.1% of the patients (*n* = 210) having moderate to extreme pain, and 65.1% of the patients (*n* = 148) were experiencing pain at least half the time on the first post-operative day. In contrast, 83.3% of the patients (*n* = 100) receiving APS care had moderate to extreme pain on the first postoperative day, and only 46.6% of the patients (*n* = 56) had pain more than half of the time, with nobody indicating to experience severe pain constantly in this group (Table 2). A larger proportion, 74.2% (*n* = 89), of patients in the APS group reported pain relief greater than 60% compared to 61.9% of the patients (*n* = 141) in the control group. In the APS group, 15% of patients (*n* = 18) had complete pain relief, while in the control group, it was only 10.6% (*n* = 24), albeit these differences were not statistically significant (*χ*^2^ = 9.475, *p* = 0.0632).

**Table 2:** Comparison of pain intensity and satisfaction with pain management in the APS and control groups

Variable		APS group (*n* = 120)	Control group (*n* = 228)	*p*
Least pain in 24 h	No pain	20.8% (25)	7.0% (16)	*χ*^2^ = 25.793 *p* < 0.0001
	Low	47.5% (57)	40.8% (93)	
	Moderate	24.2% (29)	27.6% (63)	
	Severe	7.5% (9)	18.0% (41)	
	Extreme	-	6.6% (15)	
Worst pain in 24 h	No pain	4.2% (5)	0.9% (2)	*χ*^2^ = 18.919 *p* = 0.0043
	Low	12.5% (15)	7.0% (16)	
	Moderate	30.8% (37)	21.9% (50)	
	Severe	42.5% (51)	46.9% (107)	
	Extreme	10.0% (12)	23.3% (53)	
Experiencing strong pain	Never	10.0% (12)	2.2% (5)	*χ*^2^ = 25.142 *p* < 0.0001
	Smaller part	43.4% (52)	32.9% (75)	
	Half the time	30.8% (37)	33.8% (77)	
	Larger part	15.8% (19)	23.7% (54)	
	Constantly	-	7.4% (17)	
Pain relief	No relief	0.8% (1)	1.3% (3)	*χ*^2^ = 9.475 *p* = 0.0632
	Poor	4.2% (5)	12.7% (29)	
	Medium	19.2% (23)	24.1% (55)	
	Good	59.2% (71)	51.3% (117)	
	Complete relief	15.0% (18)	10.6% (24)	
	No answer	1.6% (2)	-	

The interference of pain with physical activities and sleeping (Table 3) was reported to be significantly worse in hospitals without APS regarding activities in bed (*χ*^2^ = 21.978, *p* = 0.0006) and out of bed (*χ*^2^ = 14.341, *p* = 0.0129), but no statistically significant difference was observed regarding falling asleep (*χ*^2^ = 9.749, *p* = 0.0655) and staying asleep (*χ*^2^ = 5.983, *p* = 0.2127). In particular, the pain did not limit the movement of 16.7% of the patients in bed (*n* = 20), such as sitting up and finding a comfortable position, in the APS group, while in the control group, only 5.7% of the patients (*n* = 13) indicated the same. In contrast, we found only a slight difference between the APS group and the control group in terms of the proportion of patients, who experienced severe or extreme interference of pain with activities in bed (45%, *n* = 54 versus 46.7%, *n* = 106). Furthermore, the pain did not interfere with the physical activities out of bed among 21.7% of the patients (*n* = 26) in the APS group, while the corresponding figure was 11.5% (*n* = 26) in the control group. At the same time, severe or extreme interference was reported by 50.8% of the patients (*n* = 61) in the APS group and only by 35.3% (*n* = 80) in the control group. As far as falling asleep and staying asleep are concerned, we found no significant difference between the two groups, although the proportion of patients indicating no interference with either was higher in the APS group (25% and 21.7%, respectively) than in the control group (11.9% and 11.5%, respectively).

**Table 3:** Proportion and number of patients having pain-related interference with different physical activities and sleeping in the APS (*n* = 120) and control groups (*n* = 227)

	None		Mild		Moderate		Severe		Extreme	
	APS	Control	APS	Control	APS	Control	APS	Control	APS	Control
Activities in bed	16.7% (20)	5.7% (13)	30.0% (36)	17.6% (40)	35.0% (42)	32.2% (73)	15.0% (18)	29.1% (66)	3.3% (4)	15.4% (35)
Activities out of bed	21.7% (26)	11.5% (26)	35.0% (42)	18.1% (41)	21.7% (26)	27.8% (63)	15.8% (19)	17.2% (39)	5.8% (7)	25.4% (58)
Falling asleep	25.0% (30)	11.9% (27)	31.7% (38)	29.5% (67)	28.3% (34)	26.9% (61)	13.3% (16)	20.7% (47)	1.7% (2)	11.0% (25)
Staying asleep	21.7% (26)	11.5% (26)	36.7% (44)	27.8% (63)	24.2% (29)	26.4% (60)	16.7% (20)	18.9% (43)	0.8% (1)	15.4% (35)

The interference of pain with the mental well-being of patients was stronger in the control group than in the APS group (Table 4). Only 6.6% of the patients (*n* = 8) in the APS group felt severe or extreme anxiety due to postoperative pain, while 13.2% (*n* = 30) reported the same in the control group. In the APS group, only 14.2% of the patients (*n* = 17) reported severe or extreme levels of helplessness, while 35.3% (*n* = 80) reported so in the control group. We found a significant difference in terms of the pain causing anxiety (*χ*^2^ = 11.789, *p* = 0.0374), fright (*χ*^2^ = 13.221, *p* = 0.0161), and helplessness (*χ*^2^ = 24.420, *p* = 0.0001), but not gloom (*χ*^2^ = 6.472, *p* = 0.2127), although a higher proportion of patients reported that they had not felt depressed at all in the APS group (63.3% vs. 53.3%).

**Table 4:** Proportion and number of patients having pain-related mood and emotional changes in the APS (*n* = 120) and control groups (*n* = 227)

	None		Mild		Moderate		Severe		Extreme	
	APS	Control	APS	Control	APS	Control	APS	Control	APS	Control
Anxious	46.7% (56)	31.3% (71)	33.3% (40)	33.5% (76)	13.3% (16)	22.0% (50)	5.8% (7)	9.7% (22)	0.8% (1)	3.5% (8)
Depressed	63.3% (76)	53.3% (121)	22.5% (27)	30.4% (69)	10.8% (13)	8.4% (19)	2.5% (3)	4.8% (11)	0.8% (1)	3.1% (7)
Frightened	55.0% (66)	39.2% (31)	25.8% (31)	29.1% (66)	15.0% (18)	17.6% (40)	4.2% (5)	10.1% (23)	0.0% (0)	4.0% (9)
Helpless	35.8% (43)	18.1% (41)	27.5% (33)	26.0% (59)	22.5% (27)	20.7% (47)	12.5% (15)	24.7% (56)	1.7% (2)	10.6% (24)

Regarding the side-effects of pain treatment (Table 5), there was no significant difference between the APS and the control group in the case of itching (*χ*^2^ = 6.584, *p* = 0.1604), but the APS group achieved significantly better results in the case of nausea (*χ*^2^ = 15.240, *p* = 0.0101), drowsiness (*χ*^2^ = 26.965, *p* = 0.0001), and dizziness (*χ*^2^ = 13.980, *p* = 0.0124). In particular, 3.3% of the patients (*n* = 4) in the APS group reported severe or extreme nausea, while 7.5% of the patients (*n* = 17) did so in the control group. The frequency of severe or extreme drowsiness was three times higher in the control group with 17.6% (*n* = 40), as opposed to 5% (*n* = 6) in the APS group. Furthermore, the prevalence of severe and extreme dizziness was almost four times higher in the control group (6.2%, *n* = 14) than in the APS group (1.6%, *n* = 2).

**Table 5:** Proportion and number of patients having pain-management-related side-effects in the APS (*n* = 120) and control groups (*n* = 228)

	None		Mild		Moderate		Severe		Extreme	
	APS	Control	APS	Control	APS	Control	APS	Control	APS	Control
Nausea	82.5% (99)	63.2% (144)	11.7% (14)	22.4% (51)	2.5% (3)	7.0% (16)	3.3% (4)	5.3% (12)	0.0% (0)	2.2% (5)
Drowsiness	62.5% (75)	35.1% (80)	17.5% (21)	27.2% (62)	15.0% (18)	20.2% (46)	4.2% (5)	13.2% (30)	0.8% (1)	4.4% (10)
Itching	90.0% (108)	82.0% (187)	3.3% (4)	10.5% (24)	4.2% (5)	3.5% (8)	1.7% (2)	1.8% (4)	0.8% (1)	2.2% (5)
Dizziness	75.0% (90)	57.0% (130)	15.0% (18)	28.9% (66)	8.3% (10)	7.9% (18)	0.8% (1)	4.4% (10)	0.8% (1)	1.8% (4)

The involvement of patients in treatment decisions was not significantly different between the two groups (*χ*^2^ = 1.605, *p* = 0.8130), and the satisfaction of patients was not associated with the presence or absence of APS (*χ*^2^ = 5.781, *p* = 0.2127), either, albeit 5.8% of the patients (*n* = 13) were dissatisfied or highly dissatisfied with pain treatment in the control group, while only 1.6% of the patients (*n* = 2) reported the same in the APS group (Table 6).

**Table 6:** Comparison of pain relief and satisfaction with pain management in the APS and control groups (*N* = 348)

Variable		APS group (*n* = 120)	Control group (*n* = 228)	*p*
Participation	No participation	12.5% (15)	14.0% (32)	*χ*^2^ = 1.605 *p* = 0.8130
	Mild	11.7% (14)	12.7% (29)	
	Moderate	17.5% (21)	21.0% (48)	
	Heavy	29.2% (35)	23.4% (54)	
	Full participation	27.5% (33)	27.5% (64)	
	No answer	1.6% (2)	0.4% (1)	
Satisfaction	Extremely dissatisfied	-	1.8% (4)	*χ*^2^ = 5.781 *p* = 0.2127
	Dissatisfied	1.6% (2)	4.0% (9)	
	Intermediate	9.2% (11)	11.4% (26)	
	Highly satisfied	41.7% (50)	31.1% (71)	
	Totally satisfied	46.7% (56)	50.4% (115)	
	No answer	0.8% (1)	1.3% (3)	

As far as patient information is concerned, we found no significant difference between the two groups (*χ*^2^ = 3.480, *p* = 0.0945); however, the information received was perceived as significantly more helpful in the control group (*χ*^2^ = 10.926, *p* = 0.0423) than in the APS group (Table 7).

**Table 7:** Comparison of patient information about pain management in the APS and control groups (*N* = 348)

Variable		APS group (*n* = 120)	Control group (*n* = 228)	*p*
Informed	No	22.5% (27)	14.5% (33)	*χ*^2^ = 3.480 *p* = 0.0945
	Yes	77.5% (93)	85.1% (194)	
	No answer	-	0.4% (1)	
Usefulness of information received	No information received	22.5% (27)	14.5% (33)	*χ*^2^ = 10.926 *p* = 0.0423
	Lightly	1.6% (2)	2.6% (6)	
	Moderately	10.0% (12)	16.7% (38)	
	Highly	39.2% (47)	28.5% (65)	
	Fully	26.7% (32)	37.3% (85)	
	No answer	-	0.4% (1)	

Finally, the difference between the two groups was found to be statistically significant in terms of non-pharmacological pain management methods (*χ*^2^ = 33.795, *p* < 0.00001), with patients in the APS group using non-pharmacological pain management techniques more often. Patients in the APS group were also encouraged to use these techniques more frequently; nevertheless, this difference did not prove to be statistically significant (*χ*^2^ = 6.816, *p* = 0.0519). The respondents mentioned cooling with a cold poultice as far the most frequently applied method, followed by distraction, deep breathing, physical activity/walking, praying, and listening to music to a lesser extent, while a few other options were also specified by the patients, including finding a comfortable posture, having a conversation, playing cards and physiotherapy (Table 8).

**Table 8:** Comparison of non-pharmacologic pain management techniques used in the APS and control groups (*N* = 347)

Non-pharmacologic pain management techniques	APS group (*n* = 120)	Control group (*n* = 227)
Cold poultice	65.8% (79)	30.4% (69)
Heat treatment	0.8% (1)	0.9% (2)
Deep breathing	17.5% (21)	19.2% (18)
Distraction	17.5% (21)	12.3% (28)
Imagery or visualization	-	1.8% (4)
Massage	1.6% (2)	0.9% (2)
Meditation	1.6% (2)	3.1% (7)
Relaxation	2.5% (3)	3.1% (7)
Physical activity/walking	15.8% (19)	5.7% (13)
Hearing music	9.2% (11)	9.3% (21)
Praying	12.5% (15)	7.1% (16)
Other	1.8% (2)	2.7% (6)

## Discussion

This research aimed to explore whether surgical patients in Hungarian hospitals with APS have better outcomes in terms of postoperative pain relief. We assessed how much postoperative pain interfered with the daily activities of patients, their emotions and mental health, as well as the side effects of pain treatment, satisfaction with the care, the use of non-pharmacological treatment, and to what extent they were informed, encouraged, and involved in treatment decisions.

Our findings show that patients handled by an APS achieved better results in most of the areas of pain management (summarized in Table 9), even though Hungary has hardly any tradition in the technology with only two hospitals reporting a fully established APS previously (Lovasi et al. 2021). In particular, we found that the patients experienced lower pain intensity of shorter duration in hospitals with APS than in the control group, the impact of pain on the physical activity of patients was significantly less characteristic in the APS group, and except for gloom, they experienced negative emotions (anxiety, fright, and helplessness) less frequently. The same applies to the side effects of pain treatment except for itching, and the results were also better in terms of encouraging and using non-pharmacological pain relief techniques in the APS group. Furthermore, we also detected better results in favor of the APS group considering falling and staying asleep and the effectiveness of pharmacological and non-pharmacological pain relief, but in these cases, the two groups did not differ significantly. In addition to the aforementioned exceptions, we found no statistically significant difference between the two groups in terms of patient involvement and patient satisfaction. Finally, the control group fared better regarding patient information.

**Table 9:** Summary of significant differences found in the present study

Variable	Difference
Least pain in postoperative 24 h	Lower pain intensity scores reported in the APS group
Worst pain in postoperative 24 h	Lower pain intensity scores reported in the APS group
Experiencing strong pain in postoperative 24 h	A smaller part of the time experiencing strong pain reported in the APS group
Pain-related interference to activities in bed	Lower interference scores reported in the APS group
Pain-related interference to activities out of bed	Lower interference scores reported in the APS group
Development of pain-related anxiety	Lower scores reported in the APS group
Development of pain-related fear	Lower scores reported in the APS group
Development of pain-related helplessness	Lower scores reported in the APS group
Development of pain-management-related nausea	Lower scores reported in the APS group
Development of pain-management-related itching	Lower scores reported in the APS group
Development of pain-management-related dizziness	Lower scores reported in the APS group
The usefulness of information received about pain management	Higher scores reported in the control group
Non-pharmacologic pain-management techniques used	More patients reported using non-pharmacologic pain-management techniques in the APS group

In most cases, our results agree with the published research evidence. For instance, Lee et al. (Lee et al. 2010) found that the level of pain in rest was lower among patients, cared for by APS, while according to Gould et al. (Gould et al. 1992) pain scores measured with the visual analog scale decreased remarkably after the introduction of APS at general surgery wards. More recently published studies also confirmed that the intensity of postoperative pain among surgical patients decreased after the introduction of any type of APS (Miaskowski et al. 1999; Mitra et al. 2020; Fang et al. 2021). Regarding the impact of pain on physical mobility, Mitra et al. (Mitra et al. 2020) found that pain limited the everyday activities significantly more of patients who were treated by an APS. Several studies agree that patients cared for by APS could expect better outcomes, such as more effective pain relief, greater satisfaction, and less opioid consumption (Miaskowski et al. 1999; van Boekel et al. 2015; Said et al. 2018, Webb & Kim 2018), and the same applies to pain-induced negative emotions (Mitra et al. 2020). Our results are in agreement with previous studies, which have shown that postoperative pain-related anxiety, gloom, fear, and helplessness are less common among APS treated patients, especially if a psychologist is also part of the team (Jepegnanam et al. 2021). It is important to point out that, with the exception of gloom, the results of our APS group were significantly better in terms of mental well-being, although the studied Hungarian APS team only comprised of physicians and nurses, but no psychologist (Lovasi et al. 2021).

Furthermore, our findings show that side effects were more common among patients who received regular care, which again confirms the previously published evidence. According to the international literature, the frequency of nausea and vomiting decreased, when patients were cared for by APS (Miaskowski et al. 1999; Mitra et al. 2020; Fang et al. 2021). Itching, drowsiness, and urinary retention were also found less typical among patients handled by an APS (Miaskowski et al. 1999; Werner et al. 2002; Mitra et al. 2020). Although we have not examined opioid consumption, several studies demonstrated that APS can reduce the use of opioid medication, thereby reducing side effects and shortening hospital length of stay (Said et al. 2018, 2021; Edwards et al. 2020). Consequently, APS also represents a new opportunity to improve care for patients with chronic pain or opioid dependence (Miclescu et al. 2017).

Although our results are also encouraging regarding non-pharmacological pain relief strategies, the study highlights the lack of physiotherapists in the Hungarian APS teams (Lovasi et al. 2021), as only one patient indicated receiving help from a physiotherapist. Physiotherapists have a wide range of relevant knowledge and skills in connection with pain relief, such as individual movement therapy, transcutaneous electrical nerve stimulation, manual therapy, and massage, that could improve the physical condition, mobilization, and mental health of patients, the latter by contributing to the prevention of pain-induced negative emotions (Robinson et al. 2019; Thacker et al. 2021).

There are complex patient outcomes, like patient satisfaction, which are influenced by APS, but it is challenging to find clear causal associations (Stamer et al. 2020). For instance, it is well known that lower pain intensity does not automatically improve patient satisfaction (Werner et al. 2002). Other factors, such as proper communication, appropriate information, and involvement in decision making, play a more important role (Nielsen et al. 2012; Siu et al. 2019), and we can add comfortable ambiance and good quality nutrition, too (Stamer et al. 2020). As the APS in our research did not perform better regarding the information received by patients and the involvement of patients in pain-management-related decision making, patient satisfaction might have been affected by these. The Hungarian teams have a short history only with less developed and established task sharing and team communication (Lovasi et al. 2021), which could also explain the less favorable results regarding patient information, involvement, and satisfaction. Patient information seems to be the weak point of the system as patients traditionally turn to the ward nurses with pain relief problems and may continue to do so even in hospitals with APS. Furthermore, the APS group had a higher proportion of people with lower education levels, which might also contribute to the observed results, and the impact of the pandemic should also be mentioned (Dey & Malik 2021), which diverted the resources from regular care. This further aggravated the human resource shortages, which the Hungarian health system had already struggled with (Gaál et al. 2011, Girasek et al. 2016, 2017; Szócska et al. 2021).

Our study had some limitations. We must note first the limitations of the study design. Not all variables can be controlled in a real-life hospital environment; thus, the results might merely be associative in some cases rather than casual. Furthermore, in the absence of sufficient funding, we were not able to employ research assistants in the hospitals; therefore, the research was coordinated by head nurses locally. This might have had an influence on patient selection and data collection. The implementation of the study was made more difficult by the evolving COVID-19 pandemic, which diverted the attention and capacities of the participating institutions away from regular care, and we were not able to gather data from all of the planned hospitals. Another limitation is that we were able to include the relevant departments of a single hospital operating APS in the study, which means that data came from only one department of surgery, orthopedics, and traumatology each. Since we compared the data of the patients of county hospitals with very similar parameters, providing the same level of healthcare. Regardless, we tried to collect data on the type of surgery and the type of pain management used on an anonymized form, which would have been filled out by the competent senior nurse. Presumably due to the overload caused by the ongoing pandemic, not enough care was taken to fill out the form completely; therefore, the data was so incomplete that it prevented us from processing it.

## Conclusions

In line with the international literature, this study found several important differences in the patient outcomes of surgical cases, such as pain intensity, pain-related movement limitations, pain-induced negative emotions, side effects, and non-pharmacological pain treatment methods, in hospitals with and without APS, in favor of the latter group. In contrast, no statistically significant difference was observed regarding patient involvement and satisfaction, while patient information was found to be more useful by patients in the traditional setting.

Our results confirmed that APS is a useful technology in pain management, which delivers good results in the Hungarian hospital setting and in the Hungarian health system context, too. The established teams seem to be making good progress, but there are areas for further improvement. More attention should be paid, for instance, to work organization, so that patients are offered enough time to obtain helpful information and more opportunity to be involved in the decision-making regarding their care. Nevertheless, APS will unlikely gain further ground fast in the Hungarian hospital system, if policymakers provide little support in terms of financing, organization, and governance.

## Data Availability

The datasets used during the current study are available from the corresponding author on reasonable request.

## Abbreviations

APS: Acute Pain Service
APS-POQ: American Pain Society Patient Outcome Questionnaire
PCA: Patient-controlled analgesia
SD: Standard deviation

## References

[CR1] Ahmed A, Yasir M (2015). Role of acute pain service in optimizing postoperative pain relief in a tertiary care teaching hospital. J Pak Med Assoc.

[CR2] Benjamini Y, Hochberg Y (1995). Controlling the false discovery rate: a practical and powerful approach to multiple testing. J Roy Stat Soc Ser B.

[CR3] Borracci T, Prencipe D, Masotti A, Nella A, Tuccinardi G, Margiacchi L (2016). The experience of setting up a resident-managed Acute Pain Service: a descriptive study. BMC Anesthesiol..

[CR4] Botti M, Khaw D, Jørgensen EB, Rasmussen B, Hunter S, Redley B (2015). Cross-cultural examination of the structure of the Revised American Pain Society Patient Outcome Questionnaire (APS-POQ-R). J Pain.

[CR5] Brown C, Constance K, Bédard D, Purden M (2013). Colorectal surgery patients’ pain status, activities, satisfaction, and beliefs about pain and pain management. Pain Manag Nurs.

[CR6] Deni F, Greco M, Turi S, Meani R, Comotti L, Perotti V (2019). Acute Pain Service: a 10-year experience. Pain Pract.

[CR7] Dey S, Malik S (2021). Acute Pain Services during COVID-19: challenges and solutions. Sri Lank J Anaesth.

[CR8] Dihle A, Helseth S, Christophersen KA (2008). The Norwegian version of the American Pain Society Patient Outcome Questionnaire: reliability and validity of three subscales. J Clin Nurs.

[CR9] Edwards JM, Dollar SD, Young T, Brockopp D (2020). The role of a certified registered nurse anesthetist led acute pain service in preventing persistent postoperative opioid use. J Nurs Adm.

[CR10] Erden S, Karadağ M, Güler Demir S, Atasayar S, Opak Yücel B, Kalkan N (2018). Cross-cultural adaptation, validity, and reliability of the Turkish version of revised American Pain Society patient outcome questionnaire for surgical patients. Agri.

[CR11] Erlenwein J, Koschwitz R, Pauli-Magnus D, Quintel M, Meißner W, Petzke F (2016). A follow-up on Acute Pain Services in Germany compared to international survey data. Eur J Pain.

[CR12] Eshete MT, Baeumler PI, Siebeck M, Tesfaye M, Haileamlak A, Michael GG (2019). Quality of postoperative pain management in Ethiopia: a prospective longitudinal study. PLoS ONE.

[CR13] Fang L, Chen L, Sun H, Xu Y, Jin J (2021). The effectiveness of using a nurse-led pain relief model for pain management among abdominal surgical patients: a single-center, controlled before-after study in China. Pain Manag Nurs.

[CR14] Gaál P (2005). Benefits and entitlements in the Hungarian health care system. Eur J Health Econom.

[CR15] Gaál P, Szigeti S, Panteli D, Gaskins M, van Ginneken E (2011). Major challenges ahead for Hungarian healthcare. Br Med J..

[CR16] Girasek E, Kovács E, Aszalós Z, Eke E, Ragány K, Kovács R (2016). Headcount and FTE data in the European health workforce monitoring and planning process. Hum Resour Health.

[CR17] Girasek E, Szócska M, Kovács E, Gaál P (2017). The role of controllable lifestyle in the choice of specialisation among Hungarian medical doctors. BMC Med Educ.

[CR18] Goldberg SF, Pozek JJ, Schwenk ES, Baratta JL, Beausang DH, Wong AK (2017). Practical management of a regional anesthesia-driven acute pain service. Adv Anesth.

[CR19] Gordon DB, Polomano RC, Pellino TA, Turk DC, McCracken LM, Sherwood G (2010). Revised American Pain Society Patient Outcome Questionnaire (APS-POQ-R) for quality improvement of pain management in hospitalized adults: preliminary psychometric evaluation. J Pain.

[CR20] Gould TH, Crosby DL, Harmer M, Lloyd SM, Lunn JN, Rees GA (1992). Policy for controlling pain after surgery: effect of sequential changes in management. Br Med J.

[CR21] Hayes K, Gordon DB (2015). Delivering quality pain management: the challenge for nurses. Aorn j.

[CR22] Jepegnanam C, Bull E, Bansal S, McCarthy D, Booth M, Purser E (2021). The role of the psychologist in the inpatient pain service: development and initial outcomes. Br J Pain.

[CR23] Kersting C, Kneer M, Barzel A (2020). Patient-relevant outcomes: what are we talking about? A scoping review to improve conceptual clarity. BMC Health Serv Res.

[CR24] Lee A, Chan SK, Chen PP, Gin T, Lau AS, Chiu CH (2010). The costs and benefits of extending the role of the acute pain service on clinical outcomes after major elective surgery. Anesth Analg.

[CR25] Lovasi O, Lám J, Kósik N (2020). The role of acute pain service in postoperative pain relief. Orv Hetil.

[CR26] Lovasi O, Lam J, Schutzmann R, Gaal P (2021). Acute Pain Service in Hungarian hospitals. PLoS ONE.

[CR27] Lovasi O, Gaál P, Léber A, Lám J. A műtét utáni fájdalomcsillapítás minőségének felmérési lehetőségei: többdimenziós mérőeszközök (Options for assessing the quality of postoperative pain relief: multidimensional measurement tools). Lege Art Med. 2022;32(1–2):41–47. 10.33616/lam.32.000.

[CR28] Meissner W, Mescha S, Rothaug J, Zwacka S, Goettermann A, Ulrich K (2008). Quality improvement in postoperative pain management: results from the QUIPS project. Dtsch Arztebl Int.

[CR29] Miaskowski C, Crews J, Ready LB, Paul SM, Ginsberg B (1999). Anesthesia-based pain services improve the quality of postoperative pain management. Pain.

[CR30] Miclescu A, Butler S, Karlsten R (2017). The changing face of acute pain services. Scand J Pain..

[CR31] Mitra S, Jain K, Singh J, Jindal S, Saxena P, Singh M (2020). Does an acute pain service improve the perception of postoperative pain management in patients undergoing lower limb surgery? A prospective controlled non-randomized study. J Anaesthesiol Clin Pharmacol.

[CR32] Nielsen PR, Christensen PA, Meyhoff CS, Werner MU (2012). Post-operative pain treatment in Denmark from 2000 to 2009: a nationwide sequential survey on organizational aspects. Acta Anaesthesiol Scand.

[CR33] O’Donnell KF (2015). Preoperative pain management education: a quality improvement project. J Perianesth Nurs.

[CR34] Petti E, Scher C, Meador L, Van Cleave JH, Reid MC (2018). Can multidimensional pain assessment tools help improve pain outcomes in the perianesthesia setting?. J Perianesth Nurs.

[CR35] Pozek JJ, De Ruyter M, Khan TW (2018). Comprehensive acute pain management in the perioperative surgical home. Anesthesiol Clin.

[CR36] Radnovich R, Chapman CR, Gudin JA, Panchal SJ, Webster LR, Pergolizzi JV (2014). Acute pain: effective management requires comprehensive assessment. Postgrad Med.

[CR37] Ready LB, Oden R, Chadwick HS, Benedetti C, Rooke GA, Caplan R (1988). Development of an anesthesiology-based postoperative pain management service. Anesthesiology.

[CR38] Robinson A, McIntosh J, Peberdy H, Wishart D, Brown G, Pope H (2019). The effectiveness of physiotherapy interventions on pain and quality of life in adults with persistent post-surgical pain compared to usual care: a systematic review. PLoS ONE.

[CR39] Rockett M, Vanstone R, Chand J, Waeland D (2017). A survey of acute pain services in the UK. Anaesthesia.

[CR40] Said ET, Sztain JF, Abramson WB, Meineke MN, Furnish TJ, Schmidt UH (2018). A dedicated acute pain service is associated with reduced postoperative opioid requirements in patients undergoing cytoreductive surgery with hyperthermic intraperitoneal chemotherapy. Anesth Analg.

[CR41] Said ET, Drueding RE, Martin EI, Furnish TJ, Meineke MN, Sztain JF (2021). The implementation of an acute pain service for patients undergoing open ventral hernia repair with mesh and abdominal wall reconstruction. World J Surg.

[CR42] Sinatra R (2010). Causes and consequences of inadequate management of acute pain. Pain Med.

[CR43] Siu E, Quick JS, Xu X, Correll DJ (2019). Evaluation of the determinants of satisfaction with postoperative pain control after thoracoscopic surgery: a single-center, survey-based study. Anesth Analgesia.

[CR44] Stamer UM, Mpasios N, Stüber F, Maier C (2002). A survey of acute pain services in Germany and a discussion of international survey data. Reg Anesth Pain Med.

[CR45] Stamer UM, Liguori GA, Rawal N (2020). Thirty-five years of acute pain services: where do we go from here?. Anesth Analg.

[CR46] Subramanian P, Ramasamy S, Ng KH, Chinna K, Rosli R (2016). Pain experience and satisfaction with postoperative pain control among surgical patients. Int J Nurs Pract.

[CR47] Sun E, Dexter F, Macario A (2010). Can an acute pain service be cost-effective?. Anesth Analg.

[CR48] Szigeti S, Evetovits T, Kutzin J, Gaál P (2019). Tax-funded social health insurance: an analysis of revenue sources. Hungary Bull World Health Organ.

[CR49] Szócska M, Pollner P, Schiszler I, Joó T, Palicz T, McKee M (2021). Countrywide population movement monitoring using mobile devices generated (big) data during the COVID-19 crisis. Sci Rep.

[CR50] Tawfic QA, Freytag A, Armstrong K (2021). A survey of acute pain service in Canadian teaching hospitals. Braz J Anesthesiol.

[CR51] Thacker L, Walsh RM, Shinyoung Song G, Khan HA, Parmar P, Vance KT (2021). Exploring physiotherapy practice within hospital-based interprofessional chronic pain clinics in Ontario. Can J Pain.

[CR52] Torabi Khah M, Yousefi H, Monazami Ansari AH, Musarezaie A (2020). Prevalence of postoperative nausea and vomiting and pain in patients undergoing elective orthopaedic surgery in Iran. J Perianesth Nurs.

[CR53] Upp J, Kent M, Tighe PJ (2013). The evolution and practice of acute pain medicine. Pain Med.

[CR54] van Boekel RL, Steegers MA, Verbeek-van Noord I, van der Sande R, Vissers KC (2015). Acute pain services and postsurgical pain management in the Netherlands: a survey. Pain Pract.

[CR55] Wang H, Sherwood GD, Gong Z, Ren L, Liu H (2017). Reliability and validity of the Chinese version of the Revised American Pain Society Patient Outcome Questionnaire in postoperative patients. Pain Manag Nurs.

[CR56] Webb CAJ, Kim TE (2018). Establishing an acute pain service in private practice and updates on regional anesthesia billing. Anesthesiol Clin.

[CR57] Werner MU, Søholm L, Rotbøll-Nielsen P, Kehlet H (2002). Does an acute pain service improve postoperative outcome?. Anesth Analg.

[CR58] Zaccagnino MP, Bader AM, Sang CN, Correll DJ (2017). The perioperative surgical home: a new role for the acute pain service. Anesth Analg.

[CR59] Zoëga S, Ward S, Gunnarsdottir S (2014). Evaluating the quality of pain management in a hospital setting: testing the psychometric properties of the Icelandic version of the revised American Pain Society patient outcome questionnaire. Pain Manag Nurs.

